# Reduced hair cortisol concentrations are associated with improved emotional wellbeing in older adults following repeated forest walking

**DOI:** 10.1038/s41598-025-08378-4

**Published:** 2025-07-01

**Authors:** Daniela Jezova, Natasa Hlavacova, Lucia Karailievova, Kiki Ekiawan Lamatungga, Julia Halamova, Dhanalakshmi Tamatam, Magdalena Pichlerova, Viliam Pichler

**Affiliations:** 1 Faculty of Forestry, Technical University in Zvolen, Zvolen, Slovakia; 2 Institute of Experimental Endocrinology, Biomedical Research Centre, Slovak Academy of Sciences, Bratislava, Slovakia; 3 Institute of Applied Psychology, Faculty of Social and Economic Sciences, Comenius University, Bratislava, Slovakia; 4 Faculty of Ecology and Environmental Sciences, Technical University in Zvolen, Zvolen, Slovakia; 5 Laboratory of Pharmacological Neuroendocrinology, Institute of Experimental Endocrinology, Biomedical Research Center, Slovak Academy of Sciences, Dubravska cesta 9, Bratislava, 845 05 Slovak Republic

**Keywords:** Chronic stress, Forest environment, Older adults, Biomarkers, Endocrinology, Health care, Medical research

## Abstract

The main hypothesis of this study in older adults is that repeated walks in a forest but not an urban environment for one month lead to reduced chronic stress compared to the previous month without any intervention. This was achieved by the measurement of cumulative cortisol concentrations in hair. Older adults of both sexes (*n* = 54; 71 ± 6.2 years) participated in a randomised, parallel-group trial. They were randomly assigned to a forest or an urban walking group. They completed two 40-minute walking sessions per week over one month. Hair samples and morning, as well as afternoon salivary samples, were collected at baseline and following one month of walking interventions.

A significant reduction in cumulative hair cortisol was observed during the month of repeated forest but not urban walking compared to the previous month, indicating decreased chronic stress. Salivary cortisol concentrations decreased in the forest group only. No differences in salivary alpha-amylase activity were noticed. Walking activities had no negative impact on the diurnal rhythmicity of stress markers. Quality of life measures showed improvements in emotional well-being in the forest group. A negative correlation was found between hair cortisol and certain quality of life dimensions in urban but not forest groups. Repeated forest walks affect objective measures of chronic stress in older adults, evidenced by lower cumulative hair cortisol concentrations and improved emotional well-being. These findings encourage incorporating forest-based interventions into mental health programs for older adults aimed at enhancing well-being, stress coping, and cognitive functions.

## Introduction

For ages, human beings with an “aching heart” or a “distress in the soul” had recognised the beneficial effects of staying or walking in green spaces, particularly in forests. These can be called “antistress effects” using the nowadays terminology. Nevertheless, the objective signs and the interference of forest environments with neuroendocrine and psychological measures are still largely unknown or not sufficiently understood^1^.

Concerning stress hormone release, initial studies were based on measurements of stress hormone concentrations in blood serum or plasma. However, the blood sampling itself is inconvenient and stressful. A big progress has been enabled by the availability of the measurement of some stress markers in saliva^2^. That makes it possible a non-invasive evaluation of the two main stress systems, the hypothalamic-pituitary-adrenocortical (HPA) axis by salivary cortisol concentrations and the sympatho-adrenomedullary system (SAS) reflected by the activity of salivary alpha-amylase^3,4^. The results of the studies performed showed acute effects of short-term walking in forest environments, for example, a decrease in salivary cortisol concentrations after 15 min of walking in forested but not urban environments^5,6^. A recent meta-analysis^7^ has led to the conclusion that there is evidence of the contribution of forest therapy to urban residents’ health and well-being. According to these authors, forest therapy may reduce blood pressure and salivary cortisol concentrations on average, but may not exclude adverse results. They may include modulation of daily rhythms of the HPA axis and SAS function, however, relevant information is not available.

The results of studies investigating cortisol release following repeated and prolonged stays in forest environments are almost lacking. A recent study protocol proposing measurement of salivary cortisol concentrations and 30-minute walks in forests and urban built environments was focused on the effects of noise in this context^8^. Interestingly, a very appropriate methodological approach to evaluate chronic cortisol secretion has not yet been used in this field of research. Cortisol (and some other steroid hormones) continuously released by the adrenal cortex is cumulating in hair^9^ allowing the measurements to reflect chronic cortisol release in the preceding months. To the best of our knowledge, there is no study describing cumulative cortisol concentrations in hair comparing repeated or chronic stays in the forest compared to urban environments.

Participants in the majority of studies performed so far have been young healthy volunteers, who have many activities helping them cope with daily stress situations. In contrast, elderly people have much fewer alternatives than needed to overcome real-life stressors. Ochiai et al.^10^ observed a decrease in serum cortisol concentrations in response to relaxation and stress management activity in the forest in middle-aged males with high-normal blood pressure. However, they investigated a small sample of 9 participants and implied only one day of forest therapy. The only study evaluating salivary cortisol concentrations related to a forest therapy program in elderly participants demonstrated reduced salivary cortisol concentrations and improvement in quality of life (QoL) measures in the forest group compared to the control group^11^. A 3-day stay in the forest was combined with a cognitive behavior therapy-based intervention program, thus the effects of the forest environment alone cannot be evaluated.

The present study, performed in older adults, aimed to test the hypothesis that repeated walks in a forest but not an urban environment, conducted for one month, lead to reduced chronic stress as compared to the previous month without any intervention. This was achieved by the retrospective assessment of cumulative cortisol concentrations in hair. The secondary hypothesis tested was that repeated walks in a forest result in changes in salivary stress markers, specifically alpha-amylase activity and cortisol concentrations measured at two times of the day. The secondary hypothesis was based on the fact that a single biomarker value does not provide information on the function of the systems studied. Additionally, we anticipated a positive impact of the interventions on the overall well-being of the subjects, as assessed by QoL measures.

## Materials and methods

### Subjects

The data presented here were collected as part of a previous study investigating the effects of a repeated walking intervention over one month (two 40-minute walking sessions per week), comparing forest and urban environments, on the heart rate variability and cognitive functioning in elderly individuals^12^. The study sample consisted of older adults of both sexes residing in Zvolen, Slovakia, an urbanised and industrialised area with a population of 43,000. Participants were recruited in collaboration with municipal social services and elderly subject welfare authorities. The minimum sample size of 52 participants was calculated using GPower 3.1, with parameters set for a medium effect size (Cohen’s d = 0.5), a significance level of 0.05, and a power of 0.80. Initially, 74 elderly subjects attended the first examination, and 54 of them met the inclusion criteria. Inclusion criteria required participants to be (1) aged 60 years or older, (2) able to walk independently, even with a walking aid, and (3) committed to participating for the entire month of investigation. In this study, participants using psychotropic drugs and/or corticosteroids were explicitly excluded. This exclusion criterion was implemented to minimize potential confounding effects on neuroendocrine outcomes. The remaining medication use among participants was registered and categorized as follows: (1) Hypertension medications, (2) Cardiovascular system medications, (3) Diabetes medications, (4) Medications for pain/inflammation (joints), (5) Medications for head and back pain, (6) Osteoporosis medications, (7) Medications for reflux, (8) No medication use. Exclusion criteria included residing in institutional care. A table showing baseline demographic and clinical characteristics for each group is presented in our earlier work^12^.

### Ethics statement

This study was approved by the Ethical Committee of the Banska Bystrica Self-Governing Region for Biomedical Research, registration No. 37828100. The participants provided their written informed consent to participate in this study. For the publication of any potentially identifiable images included in this online open-access publication, written informed consent was duly obtained from the respective individual(s). The study was conducted in accordance with the ethical guidelines of the Declaration of Helsinki, as revised in 2000.

### Study design and interventions

The study was performed from the 1st to the 31st of October 2021 in Central Slovakia. It employed a randomised, parallel-group intervention design, as described in detail previously^12^. The participants were recruited in collaboration with the municipal social and older adult welfare authorities from June to August 2021. Participants who fulfilled inclusion and exclusion criteria were assigned to two groups: forest walkers as the intervention group (forest, *n* = 27) and urban walkers (urban, *n* = 27) as the active control group. The random assignment of volunteers to the two groups was performed using a web-based research randomizer (www.randomizer.org) in a 1:1 ratio through simple randomization, i.e. subjects were assigned randomly into two groups, forest and urban. Determination of whether a subject would undergo forest or urban intervention was made by reference to a statistical series based on random sampling numbers by Prof. Viliam Pichler. Subjects were informed about their allocation 4 to 2 days before the start of the intervention, when the baseline data were collected and measurements were taken. Blinding from the specific research questions was conducted for participants and the research assistants.

Baseline data were collected four to two days before the start of the study, including psychological questionnaires and a health survey. Participants were instructed to complete two interventions per week, totalling eight visits over one month. Intervention times were randomly assigned to fit participants’ daily schedules. Each participant received their intervention schedule via mobile phone at the beginning of each week and was reminded one day before each session. On the day of the walking intervention, participants were individually transported to their assigned sites and met by a site assistant who reiterated the walking instructions. The use of cell phones, eating, and casual conversation was prohibited during the intervention, although drinking water was permitted. Assistants guided the participants by walking approximately 10 m ahead without interaction during the 40-minute sessions. These sessions consisted of 25–30 min of walking at a pace of approximately 3 km/h and 10 min of observing the environment from a portable chair or bench. The two parallel interventions were identical except for the environment, i.e., forest and urban. The trial ended according to protocol when all subjects completed eight scheduled interventions, i.e., after four weeks. The participants were instructed not to perform any unusual physical activities. They were also asked to refrain from walking in the environment opposite to the one they were assigned to for the study. All participants wore Garmin Venu SQ, a consumer wrist-worn fitness tracking device using photoplethysmography, GPS, and accelerometer sensors, 24 h a day during the whole intervention period. There were no differences in the number of steps between the groups. Only two participants from the forest walking group dropped out during the intervention period due to an unaligned schedule (1) and a fall at home (1).

**Fig. 1:**
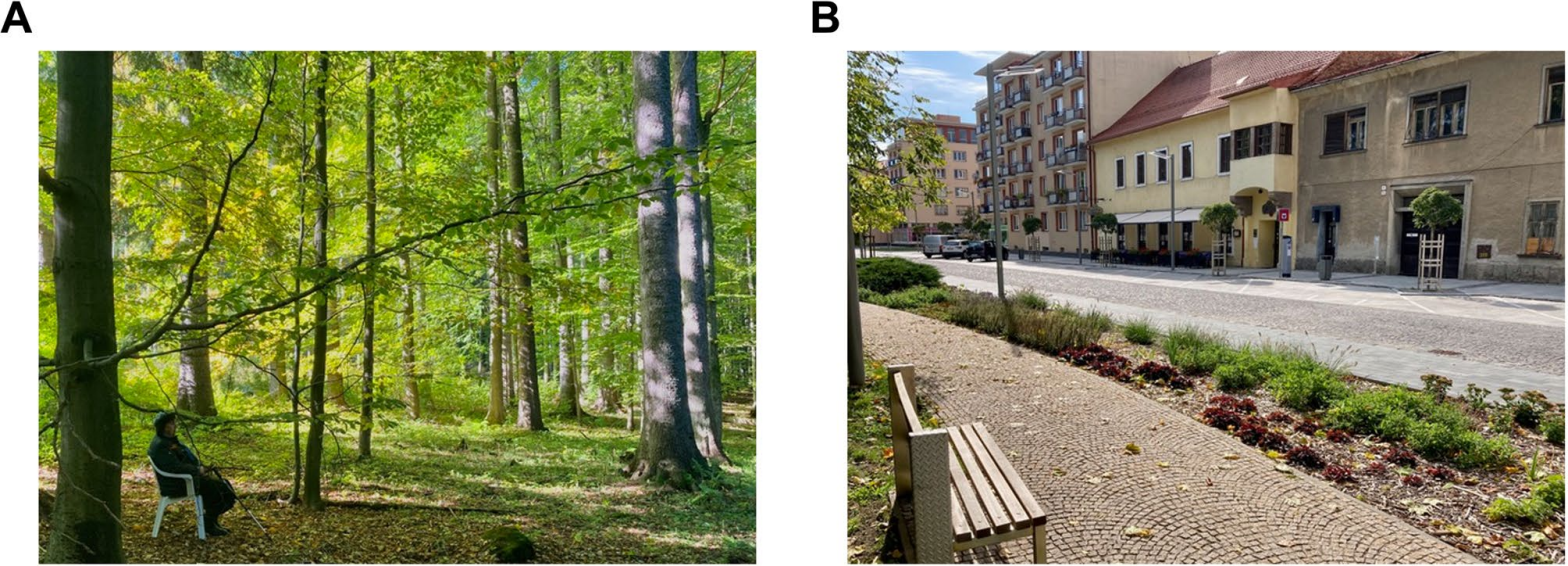
Photographs of the forest (A) and urban (B) localities used for the study interventions. Images were taken by co-author Viliam Pichler and are used with permission.

The intervention sites were carefully selected based on topographic data and field reconnaissance to ensure comfortable walking conditions on relatively even surfaces with a maximum inclination of 3° to avoid physical strain. The careful selection of multiple locations for both groups was aimed at minimizing boredom and fatigue among participants. For the forest interventions, three sites, and for the urban walking two cities were chosen. The forest sites contained semi-natural to natural forests within 15–30 min drive from the participant dwellings and comprised multiple tree species and trees of various ages, as preferred by the majority of forest visitors^13^. The key difference between forest and urban localities was the presence of closed forest canopy in the former sites and the built environment in the latter ones, as seen in Fig. 1. All experimental localities are described in detail in the paper by Lamatungga et al.^12^.

### Measurements

#### Hair cortisol

Hair samples for cortisol measurement were collected two times, at baseline (the first day of walking) and after one month of walking intervention (the last day of walking) in a dedicated university hall. Hair was carefully cut with scissors as close as possible to the scalp from a posterior vertex position. The most proximal 1 cm of hair (about 10–25 mg), which enabled the measurement of a cumulative value for cortisol secretion during the last 1 month, was used for the analysis. Hair cortisol was measured using a modification of the methodology described by Balagova and Jezova^14^. Shortly, washed and dried hair samples were pulverized with ULTRA-TURRAX Tube Drive (IKA^®^ Works, Inc., Wilmington, NC). The hair powder was eluted with 2 ml of methanol. After centrifugation, 1 ml of methanol was transferred into a new glass tube and evaporated at 50 °C in a water bath. The residue was diluted with phosphate buffer saline. Cortisol concentrations in hair extracts were measured by the Salivary Cortisol Enzyme Immunoassay (ELISA) kit (IBL International, Germany).

#### Salivary cortisol and alpha-amylase

Saliva samples were collected over two different days, initially at baseline (the first day of walking) and after a one-month walking intervention (the last day of walking). Two saliva samples were collected on each of these days, one in the morning and one in the afternoon, totalling four samples per participant. For the morning saliva collection, subjects were instructed to collect saliva samples at home before getting out of bed, 15–20 min after awakening. Saliva samples were collected into saliva sampling tubes (Salivette^®^ device; Sarstedt, UK). The participants were asked to place the cotton roll in their mouth and allow it to saturate with saliva for 1–2 min. Saliva samples were stored in a freezer at − 20 °C until analysed. Concentrations of cortisol in saliva were determined using a commercially available enzyme-linked immunosorbent assay (IBL International, Germany). The intra- and inter-assay coefficients of variation were 2.9% and 5.0%, respectively. The activity of salivary alpha-amylase was measured by a commercially available kinetic reaction assay (Salimetrics, UK). Intra- and inter-assay variance was 2.4% and 3.5%, respectively.

#### Quality of life (QoL)

To assess health-related QoL, we employed the standardized Slovak version of the 36-item Short-Form Health Survey (SF-36). The SF-36 is a widely used, validated instrument designed to evaluate overall QoL in a comprehensive yet user-friendly format. The SF-36 includes eight health scales: physical functioning (10 items), role limitations due to physical health problems (4 items), pain (2 items), general health perceptions (5 items), emotional well-being (5 items), role limitations due to emotional problems (3 items), social functioning (2 items), and energy/fatigue (4 items)^15^. These scales are aggregated into two distinct summary dimensions:​ (1) Physical health which reflects the combined scores of scales primarily related to physical health, including physical functioning, role limitations due to physical health, pain, and general health perceptions, and (2) Mental health which reflects the combined scores of scales primarily associated with mental health, encompassing emotional well-being, role limitations due to emotional problems, social functioning, and energy. Each scale is scored from 0 to 100, with higher scores indicating better perceived health and quality of life. The survey was administered at the beginning of the study and the end of the intervention period in a dedicated university hall.

#### State and trait anxiety

The Slovak version of the State-Trait Anxiety Inventory (STAI) was used for the evaluation of state and trait anxiety^16^. The questionnaire consists of two subscales specifically examining the state (STAI-X1) and trait anxiety (STAI-X2) dimensions. Both subscales contain 20 self-statements, to which participants must respond on a 1–4 scale, yielding total scores of 20–80. For the trait subscale, the subjects are instructed to have their responses based on how they generally feel, while for the state subscale, they are asked to respond how they feel at that moment. STAI-X1 was administered at the beginning of the study and after one month of walking intervention. STAI-X2 was administered only once, at the time of baseline examination in a dedicated university hall.

### Data availability section

The data that support the findings of this study are not openly available due to reasons of sensitivity and are available from the corresponding author upon reasonable request. Data are located in controlled access data storage at the Biomedical Research Center of the Slovak Academy of Sciences.

### Statistics

Data were checked for normality of distribution by the Shapiro-Wilk test. Descriptive statistics and boxplots indicated nine outlying observations (values exceeding ± 1.5 interquartile range) for both alpha-amylase and cortisol concentrations. Consequently, the data were winsorized (using 20% two-sided quantile trimming as recommended by Wilcox and Keselman^17^ before statistical modelling.

The effects of group and intervention on hair cortisol, salivary cortisol concentrations, and alpha-amylase activity, as well as on state anxiety, were evaluated by repeated measures ANOVA for factors intervention (baseline vs. completion of interventions) and group (forest vs. urban environment) with sex included as a covariate. This approach allowed us to control for potential confounding effects of sex on the outcomes. Whenever interaction reached significance, the Tukey post hoc test was performed. To evaluate changes in salivary cortisol and alpha-amylase, repeated measures ANOVA for factors time (morning vs. afternoon) and group (forest vs. urban environment) with sex included as a covariate was used. Whenever interaction reached significance, the Tukey post hoc test was performed. For the SF-36 survey data, a Mann-Whitney U test was initially used to assess differences between the two environments due to non-normal data distribution. No significant differences were found between the forest and urban groups. Subsequently, a Wilcoxon signed-rank test was performed to examine within-group comparisons before and after the intervention. To assess differences in the distribution of medication use between the urban and forest groups, chi-square tests of independence for each medication category were performed. Medication use was coded as binary (1 = use, 0 = non-use) across eight categories. All other data were analysed by a t-test for independent groups. Pearson’s correlation analyses were computed to determine relationships between hair cortisol concentrations and SF-36 scales. Results are expressed as means ± SD. The overall level of statistical significance was defined as *p* < 0.05. The statistical analyses were performed with SPSS or Statistica 9.0 statistical software.

## Results

### Characteristics of participants

Baseline characteristics are presented in Table 1. Females and males were equally distributed across both environments to mitigate confounding effects related to sex differences. The mean age was 72.3 years in the forest group and 69.7 years in the urban group (*p* = 0.055). Other characteristics, including BMI, tobacco/smoking status, and chronic illness, scores in STAI-X2 (trait anxiety) and STAI-X1 (state anxiety) did not differ significantly between the two groups.

**Table 1:** Characteristics of the study participants.

Characteristics	Group	
	Forest (*n* = 27)	Urban (*n* = 27)
Sex		
Males	10	10
Females	17	17
Age in years*	72.3 (5.2)	69.7 (4.5)
BMI*	27.94 (4.5)	27.87 (4.4)
BMI Underweight	4 (14)	4 (14)
BMI Overweight	10 (37)	8 (29)
Tobacco/Smoking status		
Non-smoker	20 (74.07)	25 (92.59)
Ex-smoker	2 (7.40)	2 (7.40)
Current smoker	5 (18.51)	–
Chronic illness		
Has 1	11 (40.74)	13 (48.14)
Has > 1	10 (37.03)	10 (37.03)
None	6 (22.22)	4 (14.81)
STAI X2* (score)	38.8 (5.89)	39.7 (8.68)
STAI X1* (score)		
Before intervention	31.2 (6.62)	29.6 (5.12)
After intervention	30.0 (6.91)	29.2 (6.45)

*Mean (SD), otherwise n (%)

The comparisons of the data on the distribution of medication use between the urban and forest groups revealed no statistically significant differences between the two groups for any medication type. All p-values were greater than 0.05, indicating similar patterns of medication use in both groups. The chi-square tests showed there results: (1) Hypertension medications (urban group *n* = 18, forest group *n* = 17, χ^2^(1) = 0.12, *p* = 0.73), (2) Cardiovascular system medications (urban group *n* = 4, forest group *n* = 6, χ^2^(1) = 0.51, *p* = 0.47), (3) Diabetes medications (urban group *n* = 2, forest group *n* = 4, χ^2^(1) = 0.76, *p* = 0.37), (4) Medications for pain/inflammation (joints) (urban group *n* = 2, forest group *n* = 2, χ^2^(1) = 0.0, *p* = 1.00), (5) Medications for head and back pain (urban group *n* = 5, forest group *n* = 3, χ^2^(1) = 0.61, *p* = 0.44), (6) Osteoporosis medications (urban group *n* = 6, forest group *n* = 7, χ^2^(1) = 0.10, *p* = 0.74), (7) Medications for reflux (urban group *n* = 2, forest group *n* = 3, χ^2^(1) = 0.28, *p* = 0.59), (8) No medication use (urban group *n* = 1, forest group *n* = 0).

### Hair cortisol concentrations

**Fig. 2:**
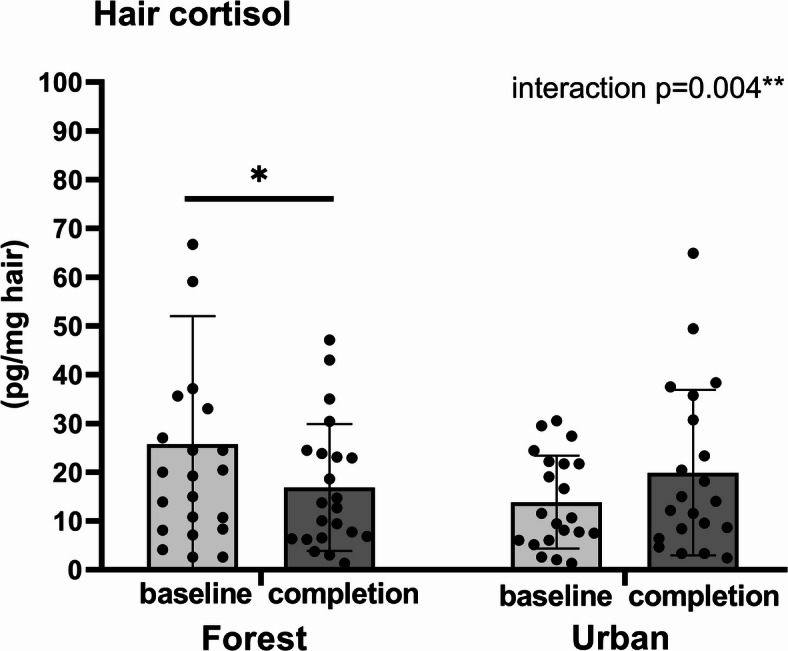
Cumulative hair cortisol concentrations before and after the completion of walking intervention in forest (*n* = 27) and urban (*n* = 27) walkers. Results are expressed as means ± SD. Statistical significance as revealed by ANOVA for repeated measures with group (forest vs. urban environment) as the between-subjects factors and time (baseline vs. completion) as the within-subjects factor, with subsequent Tukey post hoc test: *p < 0.05.

To test the primary hypothesis that repeated walks in a forest but not in an urban environment lead to reduced chronic stress, one-month cumulative cortisol concentrations were measured in hair. ANOVA for repeated measures did not reveal any significant main effect of factors group and time on hair cortisol concentrations (Fig. 2). However, a significant time x group interaction was revealed (F_(1, 41)_ = 7.42, *p* < 0.01, η^2^ = 0.838). Tukey post hoc tests indicated that cumulative hair cortisol concentrations were significantly lower during one month of walking in the forest (*p* < 0.05), but not in the urban walking group, compared to values corresponding to cortisol secretion during the month preceding the walking activities. The covariate sex was not found to be statistically significant.

### Salivary cortisol concentrations and alpha-amylase activity

**Fig. 3:**
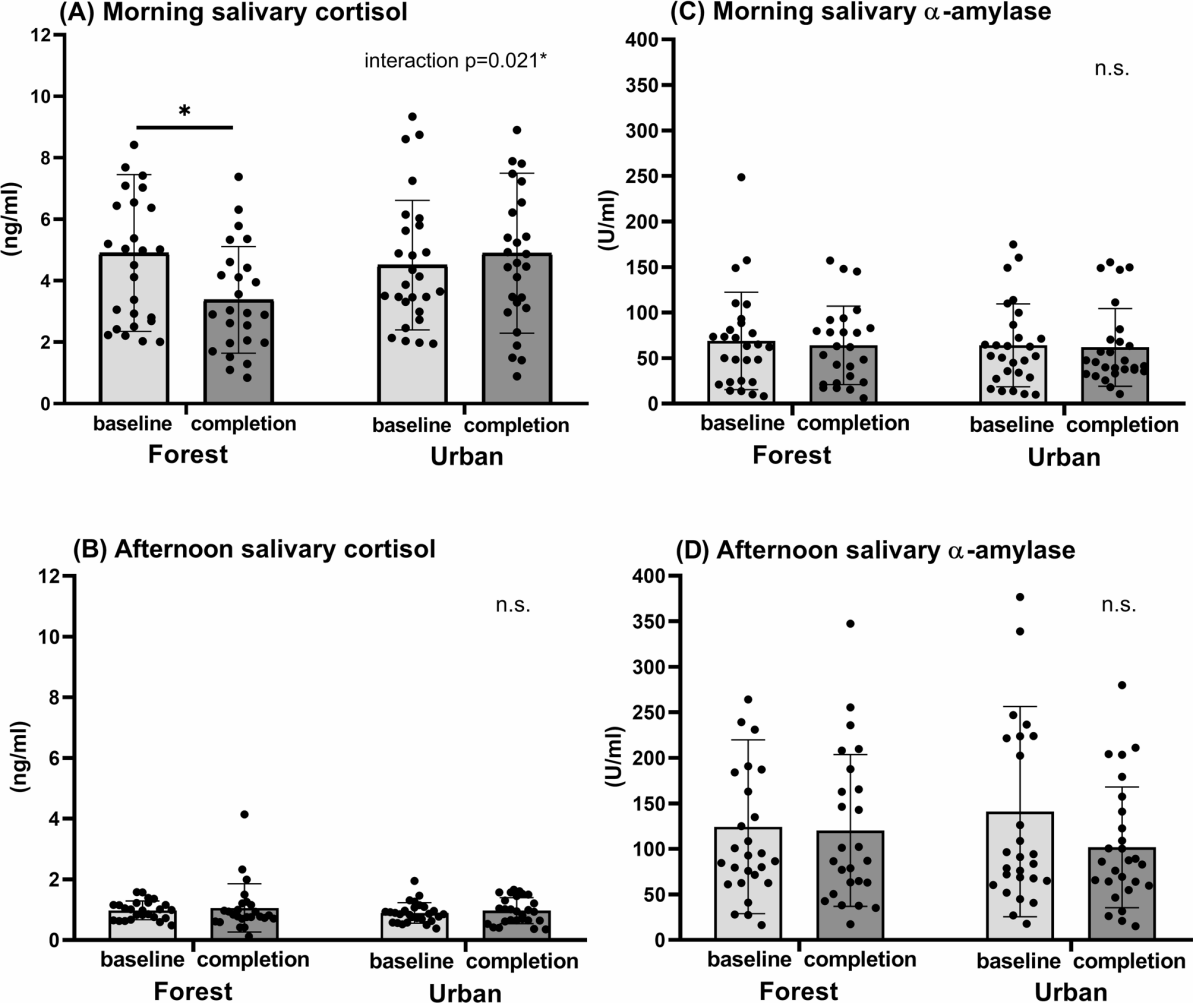
Morning (A) and afternoon (B) salivary cortisol concentrations as well as in morning (C) and afternoon (D) salivary alpha-amylase activity before and after the completion of walking intervention in forest (*n* = 27) and urban (*n* = 27) walkers. Results are expressed as means ± SD. Statistical significance as revealed by ANOVA for repeated measures with group (forest vs. urban environment) as the between-subjects factors and time (baseline vs. completion) as the within-subjects factor, with subsequent Tukey post hoc test: *p < 0.05.

To test the secondary hypothesis that repeated walks in a forest in older adults result in changes in salivary stress markers, we measured morning and afternoon alpha-amylase activity and cortisol concentrations. Repeated-measures ANOVA revealed a significant effect of time of day on salivary cortisol concentrations and alpha-amylase activity. Morning salivary cortisol concentrations (Fig. 3A, B) were significantly higher than afternoon levels in both groups at baseline (F_(1, 52)_ = 157.3, *p* < 0.001, η^2^=0.991) and the time of completion of the interventions (F_(1, 50)_ = 104.7, *p* < 0.001, η^2^=0.860. Similarly, salivary alpha-amylase activity (Fig. 3C, D) was significantly higher in the afternoon compared to the morning in both groups at baseline (F_(1, 52)_ = 22.9, *p* < 0.001, η^2^=0.994) and after the interventions (F_(1, 50)_ = 12.6, *p* < 0.001, η^2^=0.773).

No significant main effects of group or intervention on morning salivary cortisol concentrations (Fig. 3A) were found. However, a significant group x time interaction was observed (F_(1, 50)_ = 8.06, *p* < 0.01, η^2^ = 0.496). Tukey post hoc tests showed that morning salivary cortisol concentrations were significantly lower following the completion of a one-month walking intervention in the forest group (*p* < 0.05), but not in the urban environment group. There were no significant main effects of factors group and intervention or a significant interaction between these factors on afternoon cortisol concentrations measured in saliva (Fig. 3B). ANOVA for repeated measures did not show significant main effects of group or time on salivary alpha-amylase activity measured in the morning and afternoon (Fig. 3C, D). No significant interaction between factors was observed. The covariate sex did not show a significant effect on salivary cortisol concentrations and alpha-amylase activity.

### QoL

To determine the expected positive impact of the interventions on the overall well-being of the subjects, QoL measures were evaluated. Although no significant differences were observed between the subjects walking in the forest and urban environments for SF-36 outcomes, the overall median scores of the SF-36 indicated a good QoL (Table 2). Two components of mental health showed significant improvement: emotional well-being (Z=-3.169, *p* < 0.01, *r* = 0.611) in the forest group and energy (Z=-2.515, *p* < 0.05, *r* = 0.484) in the urban group. However, general health perception, a sub-component of physical health, decreased by 5 points in the forest environment (Z=-2.035, *p* < 0.05, *r* = 0.392).

**Table 2:** Median scores of SF-36 components between forest and urban environments before and after interventions.

Components		Forest									Urban										
		Before		After		Z		*p*			Before		After		Z			*p*			
Physical health																					
Physical functioning		80		80		–0.132		0.895			90		85		–0.024			0.981			
Role function. physical		62.5		68.75		–1.651		0.099			71.87		75		–1.23			0.216			
Pain		67.5		67.5		–1.495		0.135			77.5		77.5		–0.770			0.441			
General health		55		50		–2.035		**0.042**			55		55		–0.820			0.412			
Mental health																					
Emotional well-being		75		85		–3.169		**0.002**			77.5		80		–1.953			0.051			
Role function. emotional		75		66.66		–0.432		0.665			75		75		–0.696			0.487			
Social functioning		75		87.5		–1.395		0.163			75		87.5		–1.147			0.251			
Energy		68.75		62.5		–1.673		0.094			68.75		75		–2.515			**0.012**			

### Correlation analyses

**Fig. 4:**
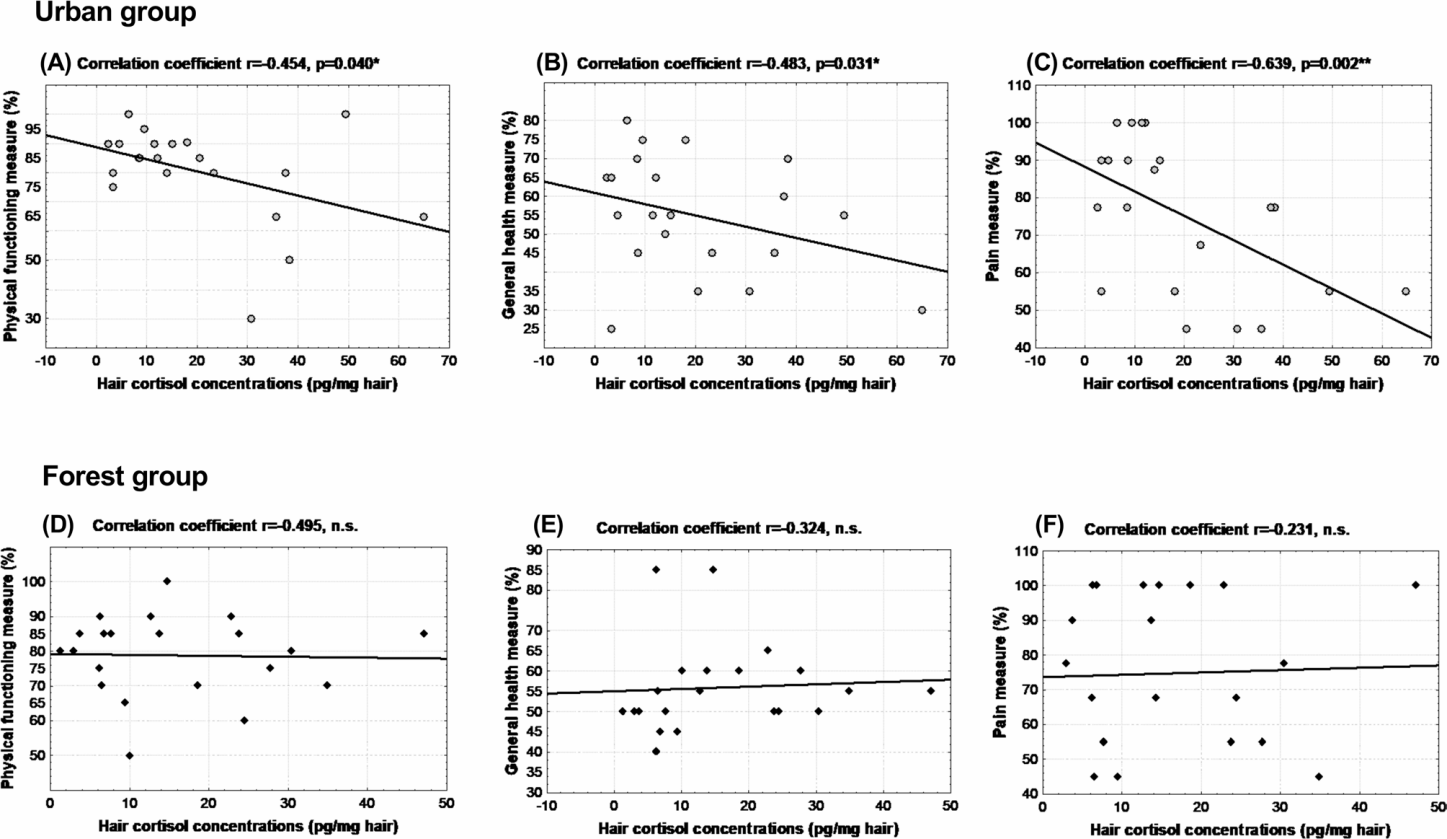
Correlations between SF-36 health survey measures and hair cortisol concentrations measured at the end of walking interventions, separately in the forest and urban group. Statistical significance as revealed by Pearson correlation.

Pearson correlation analysis was used to test the hypothesis that there are associations between SF-36 health survey measures and hair cortisol concentrations measured at the end of one-month walking interventions. Correlation analyses were performed separately in the forest and urban groups (Fig. 4, A-F). In the urban walking group, significant negative correlations were found between hair cortisol concentrations and measures of the SF-36 health survey, specifically physical functioning (*r*=-0.454, *p* < 0.05), pain (*r*=-0.639, *p* < 0.01), and general health (*r*=-0.484, *p* < 0.05). These associations were absent in participants who walked in a forest environment.

## Discussion

The main finding of the present study is that cumulative secretion of the stress hormone cortisol measured in hair in older adults was significantly lower during the month of repeated forest but not urban walking compared to the previous month without walking activities. Emotional well-being, but not the other measures of the SF-36 Health Survey in elderly subjects, was improved following repeated walks in a forest compared to walks in an urban environment.

The use of hair cortisol as a chronic stress indicator in the research field of nature-based interventions is still scarce. To the best of our knowledge, no papers focused on the effect of individual long-term forest interventions and measurement of hair cortisol are available, making our study distinctive. We have revealed reduced cumulative cortisol concentrations in older adults performing repeated walks in a forest but not in an urban environment, thus confirming the beneficial influence of natural forest stays. This finding is supported by the results of a marginally related study showing lower hair cortisol concentrations in people living in areas with natural environments^18^. However, cumulative cortisol concentrations in hair did not differ in individuals undergoing mindfulness-based stress reduction interventions^19^ or physical exercise^20^ in different environments. The mentioned reports were based on a variety of natural environments and included participants of various ages. Clark et al. (2023) emphasized the need to identify and report forest features that most positively affect mental health and subjective well-being. Pichlerová et al.^22,23^ highlighted age-dependent patterns in forest visit frequency and psycho-emotional responses.

Morning salivary cortisol concentrations observed in the present study in older adults decreased significantly following repeated forest but not urban walks. This finding is consistent with the reduced cumulative cortisol concentrations in hair, although salivary cortisol reflects only the current hormone concentrations at the time of sampling. Similar studies on repeated forest walking in elderly participants are scarce. Long-term nature experience (3 times a week for 8 weeks) with a non-standardized design in middle-aged male subjects led to a mild modification of the salivary cortisol diurnal profile^24^. The current results are in line with a decrease in salivary cortisol concentrations after short-term walking in forested but not urban environments in young individuals, e.g^5,6^. A 4-hour forest therapy in women aged 40–73 years resulted in a decrease in salivary cortisol concentrations^10^. The present finding is not in line with the data of Toda et al.^25^, who investigated the impact of short walking in woodland on cortisol concentration in 20 older adults and found that cortisol levels remained unchanged after walking. However, the walk included an elevation gain of 260 m as opposed to a maximum of 40 m in the present study. Thus, the walk was rather demanding for elderly participants, and the physical stress might have prevented the expected decrease in salivary cortisol induced by forest ambience.

Repeated walking in both forest and urban environments did not disturb the physiological differences between morning and afternoon cortisol secretion and salivary alpha-amylase activity. The daily rhythms of cortisol secretion and salivary alpha-amylase activity are opposite, with the highest levels of cortisol and the lowest activity of alpha-amylase in the morning^26,27^. Both morning and afternoon values of alpha-amylase activity did not differ between forest and urban groups investigated in the present study. Nater et al.^28^ showed that salivary alpha-amylase activity follows a distinct diurnal profile. The daily pattern is thought to reflect autonomic regulatory processes rather than direct catecholamine secretion. Apparently, long-term changes in autonomic nervous system activation are better reflected by other measures, as shown by the induction of higher heart rate variability as a parasympathetic activity marker in older adults exposed to repeated forest walks in our sample of subjects^12^.

In the present study, emotional well-being but not the other measures of the SF-36 Health Survey in elderly participants were improved following repeated walks in the forest compared to walks in the urban environment. We did not find a significant difference in overall QoL between the two investigated environments. Improvements in different categories of SF-36 were observed in the forest compared to the urban walking group. Emotional well-being was significantly improved after the intervention in the forest walkers, while energy or vitality was significantly improved from baseline to study completion in the urban group. We speculate that the increased emotional well-being of forest walkers could result from frequent feelings of freedom and gratitude, which were often reported by people visiting forests during the COVID-19 pandemic^23^. Appreciation and gratitude as positive emotions play a causal role in fostering well-being, possibly by reducing hedonic adaptation^29^. One possible explanation for the increased energy and vitality observed in the urban group is that the usual presence of other people in public spaces could foster relational restoration, as conceptualized by Hartig^30^. In contrast, general health, as part of the physical health component, declined in forest walkers compared to urban walkers. This decline could be due to walking conditions in forests, such as uneven terrain and colder temperatures, which might temporarily impact how people assess their general health. The apparent contradiction between the present observation of significant improvement in emotional well-being with a concurrent decline in physical health in the forest group can be explained by only a small correlation between emotional well-being and physical health^31^.

The present results of correlation analyses in the urban group showing negative associations between physical functioning, pain, and general health measures as QoL components and hair cortisol concentrations as an objective stress level marker come as additional novel findings. They have the potential to shed new light on stress as a mediator between nature exposure and QoL, whose role as such was suggested by Bacevicienė and Jankauskienė^32^ based on multiple standardized scale questionnaires. The correlations described above were not statistically significant in the forest group. However, the negative correlations remained statistically significant for physical functioning and pain, when the correlation analysis was performed in all participants irrespective of intervention group. The significant negative correlations between hair cortisol concentrations and several SF-36 health survey measures in the urban group but not in the forest group suggest differential impacts of the two environments on stress and health. The possibility that forest walking could weaken the association between hair cortisol concentrations and pain, physical functioning, and overall health is plausible given the multiple pathways through which contact with nature can improve health. These pathways include not only stress reduction^33^, but also enhanced immune function due to factors such as soil bacteria, phytoncides, and others^34^. For instance, Mycobacterium vaccae NCTC 11,659, a common soil-derived bacterium was shown to have anti-inflammatory, immunoregulatory, and stress-resilience properties^35^. Environmental microorganisms may induce such effects following their inhalation^36^, e.g. after their release during forest walking that normally disturbs the soil surface or soil litter layer. In contrast, except for relational restoration, these benefits are not inherent to urban built environments.

## Strengths and limitations

A key strength of this study is the use of hair cortisol concentration as an objective biomarker for chronic stress, offering a reliable measure of long-term stress regulation. This adds a robust, quantifiable element to the research, enhancing its relevance for assessing physiological stress adaptation. Additionally, the study’s novel design, which prevented relational restoration and included exposure to elements of a primaeval forest, allowed for a focused evaluation of nature’s direct impact on chronic stress coping, independent of social interactions. Isolating the effects of nature exposure in this way, particularly within a primaeval forest, provides a clearer understanding of its unique role in stress recovery. However, the sample size, limited to older adults in Slovakia, partly restricts the generalizability of the findings.

## Conclusions

The results of this carefully designed study demonstrate that repeated walks in a forest environment for one month affect objective measures of chronic stress in older adults. The observed changes in stress hormone release suggest a possible contribution to the beneficial influence of the forest environment on human health and well-being. Based on the data of the present study, as well as those published previously^12^, it can be recommended that healthcare institutions consider the inclusion of stays in forest environments in programs for older adults aimed at improving their well-being, stress coping, and cognitive functions.

## Data Availability

The data that support the findings of this study are not openly available due to reasons of sensitivity and are available from the corresponding author upon reasonable request. Data are located in controlled access data storage at Biomedical Research Center of the Slovak Academy of Sciences.
